# Cost-Effectiveness Thresholds for Venous Thromboembolism Prophylaxis in Ankle Fracture Surgery: A Break-Even Analysis

**DOI:** 10.1177/24730114251363497

**Published:** 2025-08-20

**Authors:** Kush Mody, Avani A. Chopra, David Ahn, Michael Aynardi, Sheldon Lin

**Affiliations:** 1 Rutgers New Jersey Medical School, Newark, NJ, USA; 2 Penn State College of Medicine, Hershey, PA, USA

**Keywords:** ankle fracture surgery, cost-effectiveness, venous thromboembolism chemoprophylaxis

## Abstract

**Background:**

The role of venous thromboembolism (VTE) chemoprophylaxis following ankle fracture surgery remains controversial. Although pharmacologic prophylaxis is standard in major orthopaedic procedures, its utility in foot and ankle trauma surgery is unclear because of low reported VTE rates and potential bleeding risks. Furthermore, no consensus exists on the cost-effectiveness of prophylactic agents in this population.

**Methods:**

A literature review and the TriNetX Research Network were used to identify postoperative symptomatic VTE rates following ankle open reduction internal fixation (ORIF). The cost of treating a symptomatic VTE was estimated from existing literature and adjusted to 2025 US dollars. Drug pricing data were obtained from an online pharmacy database. A break-even analysis was conducted to calculate the absolute risk reduction (ARR) and number needed to treat (NNT) for each agent to be cost-effective. A subanalysis compared 30-day bleeding and transfusion rates between patients who received prophylaxis and those who did not.

**Results:**

The low and high literature-based VTE rates were 0.33% and 1.2%, whereas the TriNetX-derived VTE rate was 0.56%. Among 64 184 patients undergoing ankle ORIF without prophylaxis, 384 developed a symptomatic VTE. Aspirin (81 mg and 325 mg) and warfarin (5 mg) were cost-effective at all 3 VTE rates, with NNTs ranging from 9217 to 10 547. Enoxaparin (40 mg) was only cost-effective at the highest VTE rate (NNT = 131), whereas rivaroxaban (20 mg) was not cost-effective at any rate. Enoxaparin and rivaroxaban became cost-effective only when VTE treatment costs exceeded $50 000 and $1 500 000, respectively. Patients receiving prophylaxis had higher bleeding (0.56% vs 0.26%) and transfusion (0.82% vs 0.25%) rates (*P* < .001).

**Conclusion:**

In summary, this study found that aspirin 81 mg, aspirin 325 mg, and warfarin are cost-effective for VTE chemoprophylaxis following ankle fracture fixation. Enoxaparin and rivaroxaban are generally not cost-effective, and their use may be appropriate only in high-risk patients.

**Level of Evidence:**

Level IV, economic analysis.

## Introduction

Venous thromboembolism (VTE) is a serious postoperative complication that includes deep vein thrombosis (DVT) and pulmonary embolism (PE), both of which can lead to significant morbidity and mortality. Although the risk of VTE following major orthopaedic procedures such as total hip and knee arthroplasty is well established, its incidence after foot and ankle surgery—particularly ankle fracture fixation—remains less clearly defined. Reported rates vary widely, with some studies citing incidences as high as 5.09% in nonweightbearing casted patients and others reporting rates as low as 0.42% following elective procedures.^6,15^

The utility of pharmacologic VTE chemoprophylaxis in foot and ankle trauma surgery is still debated. The American Orthopaedic Foot & Ankle Society (AOFAS) has acknowledged the current lack of high-quality evidence to either support or refute routine prophylaxis in these patients. Furthermore, existing clinical guidelines, such as the 2012 CHEST recommendations, suggest that VTE chemoprophylaxis may be unnecessary for patients with isolated lower extremity injuries requiring immobilization.^5^ Despite this, up to 98% of foot and ankle surgeons report using chemoprophylaxis in high-risk patients, underscoring the gap between clinical practice and guideline consensus.^16^

This study aims to apply a break-even cost-effectiveness analysis to determine whether commonly used pharmacologic agents for VTE prophylaxis, including aspirin, warfarin, enoxaparin, and rivaroxaban, are financially justifiable in patients undergoing operative fixation of ankle fractures. By incorporating current drug pricing, literature-based VTE rates, and real-world incidence data derived from a large electronic health record database, this analysis seeks to clarify under what conditions, if any, prophylaxis strategies are cost-effective in this patient population.

## Methods

**Figure 1.**
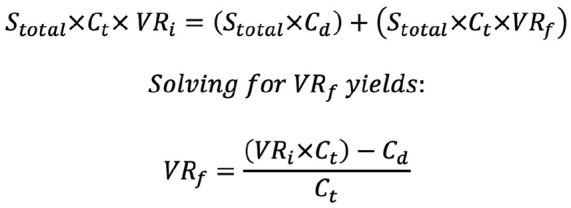
Equation for break-even cost-effectiveness model. Adapted from Hatch et al.^8^ C_d_, cost of drug; C_t_, cost of treating symptomatic venous thromboembolism; S_total_, total annual surgical procedures; VR_f_, final break-even rate; VR_i_, initial venous thromboembolism rate.

This study used a modified cost-effectiveness equation initially proposed by Hatch et al^8^ to calculate the break-even VTE rate required for a prophylactic intervention to be cost-effective (Figure 1). The break-even rate was subtracted from the initial VTE rate to determine the absolute risk reduction (ARR) needed for cost neutrality. The ARR was then used to calculate the number needed to treat (NNT), representing the number of ankle open reduction internal fixation (ORIF) surgeries that would need to be performed with prophylaxis to prevent 1 symptomatic VTE event while remaining cost-effective. If the calculated ARR exceeded the initial VTE rate, the intervention was considered not cost-effective. Additionally, sensitivity analysis was performed to assess the robustness of the model.

To estimate the financial impact of treating a symptomatic VTE, a review of published literature was conducted. The most widely cited estimate, derived from Grosse et al,^7^ reported a cost of $15 000 in 2014 USD for treating acute VTE. This figure represents the additional health care costs associated with treating an acute VTE, based on payer or health care sector perspectives. The estimate includes costs calculated either by the value of resources used or by using average payments as a proxy. This value was adjusted for inflation to $20 461.32 in 2025 USD.^4^

Drug pricing data were obtained from an online database (GoodRx; GoodRx Holdings, Santa Monica, CA) that aggregates average retail prices for prescriptions. Average costs for a 1-month supply of commonly used once-daily VTE prophylactic agents were obtained. We selected a 1-month prophylaxis duration based on the recommendation for major orthopaedic procedures such as total hip or knee arthroplasty, given that the American College of Chest Physicians guideline advises against routine VTE prophylaxis for isolated lower-leg injuries requiring immobilization. The treatments evaluated in this study include aspirin (81 mg and 325 mg), warfarin (5 mg), enoxaparin (40 mg), and rivaroxaban (20 mg). As warfarin requires regular INR monitoring, which may range in cost from $6.19 to $145.70 per test, an additional analysis was conducted using the lowest reported monitoring cost to reflect a conservative estimate.^3^ It is important to note that the reported cost of INR testing reflects only the direct cost of the test itself and does not account for indirect costs such as time off work, transportation, or other logistical burdens for patients. Although these factors are important to consider in a comprehensive cost-effectiveness analysis, they are beyond the scope of this study, which focuses solely on direct medication costs.

A literature search was performed to identify published rates of symptomatic VTE following ankle ORIF. Additionally, the TriNetX Research Network (TriNetX, Cambridge, MA), a federated network of deidentified electronic health records from more than 140 million patients across 106 health care organizations, was queried to determine the real-world incidence of VTE in patients undergoing ankle ORIF without pharmacologic prophylaxis. Patients were identified using *International Classification of Diseases, Tenth Revision* (*ICD-10*) and *Current Procedural Terminology* (*CPT*) codes associated with ankle ORIF (*CPT*: 27822, 27823, 27826, 27827, 27828, 27766, 27769, 27792, 27814, 27829) that were performed between February 2005 and February 2025. VTE events occurring within 1 month of surgery were identified using diagnosis codes for PE (*ICD-10*: I26) and lower extremity DVT (*ICD-10*: I82.4). Patients with a prescription for VTE prophylaxis within 1 month after surgery were excluded. Three initial VTE rates were selected for the cost-effectiveness analysis: 2 from the literature and 1 derived from the TriNetX query. Demographic characteristics of the study population are summarized in Table 1.

**Table 1.** Demographics for TriNetX Patients.

Characteristic	Mean or n (%)
Mean age, y	40.4
Sex, n (%)	
Male	32 741 (48)
Female	33 935 (50)
Unknown	1048 (2)
Ethnicity, n (%)	
Hispanic or Latino	11 890 (18)
Not Hispanic or Latino	40 918 (60)
Unknown	14 916 (22)
Race, n (%)	
White	46 196 (68)
Black or African American	9025 (13)
Asian	1682 (2)
Native Hawaiian or Other Pacific Islander	513 (1)
American Indian or Alaska Native	469 (1)
Other race	2987 (4)
Unknown	6852 (10)

A matched cohort subanalysis was conducted to compare the risk of 30-day postoperative transfusion and bleeding events, including intracranial hemorrhage (*ICD-10* codes: I60, I61, I62.0) and gastrointestinal hemorrhage (*ICD-10*: K92.2), between patients who received VTE chemoprophylaxis and those who did not. Patients were matched in a 1:1 ratio using greedy nearest-neighbor matching with a caliper of 0.1 pooled standard deviations, and propensity scores were estimated using logistic regression. The covariates included in the matching process were age, sex, use of systemic contraceptive, history of VTE, pregnancy, and select comorbidities (Supplemental Table 1). Matching was conducted with a tolerance of 0.01 to minimize discrepancies in propensity scores between matched pairs. Before matching in the subanalysis, 76 340 patients received VTE chemoprophylaxis, and 67 584 patients did not. After matching in the subanalysis, there were 57 054 patients in each cohort.

This study was determined to be exempt from institutional review board (IRB) approval. All data used were deidentified and derived from secondary sources. No direct interaction or intervention with human subjects occurred. This meets the criteria for exemption under §164.514(a) of the HIPAA Privacy Rule.

## Results

The product costs for a 1-month supply of once-daily aspirin (81 mg), aspirin (325 mg), warfarin (5 mg), enoxaparin (40 mg), and rivaroxaban (20 mg) were found to be $1.94, $2.00, $2.22, $156.00, and $526.22, respectively (Table 2). The lowest cost of INR testing was estimated to equal $6.19, so the total price for warfarin (5 mg) plus INR testing was $8.41. These were the lowest prices for a full month’s once-daily supply that a patient could obtain if they used GoodRx. The cost of treating a symptomatic VTE was estimated to equal $20 461.32 based on existing literature.^7^ The low and high rates of symptomatic VTE obtained from the literature were determined to be 0.33% and 1.2%.^2,14^ Using the TriNetX research database, a total of 64 184 patients underwent ankle ORIF and did not receive chemoprophylaxis. Of these patients, 384 (384/64 184 = 0.56%) were identified with a symptomatic VTE.

**Table 2.** Cost-Effectiveness of Chemoprophylactic Agents at Varying Initial Venous Thromboembolism Rates.

Drug	Cost of Drug, $	Cost of Treating VTE, $	Initial VTE Rate, %	Final VTE Rate, %	ARR, %	NNT
Aspirin (81 mg)	1.94	20 461.32	0.3	0.32	0.01	10 547
	1.94	20 461.32	0.6	0.59	0.01	10 547
	1.94	20 461.32	1.2	1.19	0.01	10 547
Aspirin (325 mg)	2.00	20 461.32	0.3	0.32	0.01	10 231
	2.00	20 461.32	0.6	0.59	0.01	10 231
	2.00	20 461.32	1.2	1.19	0.01	10 231
Warfarin (5 mg)	2.22	20 461.32	0.3	0.32	0.01	9217
	2.22	20 461.32	0.6	0.59	0.01	9217
	2.22	20 461.32	1.2	1.19	0.01	9217
Warfarin (5 mg) + INR	8.41	20 461.32	0.3	0.29	0.04	2433
	8.41	20 461.32	0.6	0.56	0.04	2433
	8.41	20 461.32	1.2	1.16	0.04	2433
Enoxaparin (40 mg)	156.00	20 461.32	0.3	−0.43	0.76	131
	156.00	20 461.32	0.6	−0.16	0.76	131
	156.00	20 461.32	1.2	0.44	0.76	131
Rivaroxaban (20 mg)	526.22	20 461.32	0.3	−2.24	2.57	39
	526.22	20 461.32	0.6	−1.97	2.57	39
	526.22	20 461.32	1.2	−1.37	2.57	39

Abbreviations: ARR, absolute risk reduction; INR, international normalized ratio; NNT, number needed to treat; VTE, venous thromboembolism.

At the product cost obtained, aspirin 81 mg was found to be cost-effective at the low, TriNetX, and high rate of symptomatic VTE if the initial rate decreased by an ARR of 0.01% (NNT = 10 547). Aspirin 325 mg was also cost-effective at all 3 initial rates with an ARR 0.01% (NNT = 10 231). Likewise, warfarin (5 mg) was cost-effective at all 3 initial rates with an ARR of 0.01% (NNT = 9217). Additionally, warfarin remained cost-effective when factoring in the lowest cost of INR monitoring (NNT = 2433) (Table 2). In contrast, cost-effectiveness was eliminated at the low and TriNetX symptomatic VTE rate for enoxaparin (40 mg) because the final VTE rate exceeded the initial rate. However, cost-effectiveness was achieved for enoxaparin (40 mg) at the high symptomatic VTE rate with an ARR of 0.76% (NNT = 131). Similarly, rivaroxaban (20 mg) was not cost-effective at the low, TriNetX, and high symptomatic VTE rate.

Sensitivity analyses demonstrated that enoxaparin (40 mg) becomes cost-effective only when VTE treatment costs exceeded $50 000 (NNT: 321) or if the VTE rate was greater than 1% (NNT: 121) (Tables 3 and 4). Similarly, analyses demonstrated that rivaroxaban (20 mg) becomes cost-effective only when VTE treatment costs exceeded $100 000 (NNT: 2851) or the VTE rate is greater than 3% (NNT: 39) (Tables 3 and 4). Additionally, aspirin and warfarin would no longer break even if VTE treatment costs are less than $500 or if the VTE rate is less than 0.001% (Tables 3 and 4). Drug cost sensitivity analysis revealed that at a VTE treatment cost of $20 461.32 and a VTE rate of 0.6%, drugs that cost greater than $100 are not cost-effective in this model (Table 5).

**Table 3.** Sensitivity Analysis for Venous Thromboembolism Treatment Cost.

Drug	Cost of Drug, $	Cost of Treating VTE, $	Initial VTE Rate, %	Final VTE Rate, %	ARR, %	NNT
Aspirin (81 mg)	1.94	100	0.6	−1.34	1.94	52
	1.94	500	0.6	0.21	0.39	258
	1.94	1000.00	0.6	0.41	0.19	515
	1.94	20 461.32	0.6	0.59	0.01	10 547
	1.94	50 000.00	0.6	0.60	0.00	25 773
	1.94	100 000.00	0.6	0.60	0.00	51 546
Aspirin (325 mg)	2.00	100	0.6	−1.40	2.00	50
	2.00	500	0.6	0.20	0.40	250
	2.00	1000.00	0.6	0.40	0.20	500
	2.00	20 461.32	0.6	0.59	0.01	10 231
	2.00	50 000.00	0.6	0.60	0.00	25 000
	2.00	100 000.00	0.6	0.60	0.00	50 000
Warfarin (5 mg)	2.22	100	0.6	−1.62	2.22	45
	2.22	500	0.6	0.16	0.44	225
	2.22	1000.00	0.6	0.38	0.22	450
	2.22	20 461.32	0.6	0.59	0.01	9217
	2.22	50 000.00	0.6	0.60	0.00	22 523
	2.22	100 000.00	0.6	0.60	0.00	45 045
Warfarin (5 mg) + INR	8.41	100	0.6	−7.81	8.41	12
	8.41	500	0.6	−1.08	1.68	59
	8.41	1000.00	0.6	−0.24	0.84	119
	8.41	20 461.32	0.6	0.56	0.04	2433
	8.41	50 000.00	0.6	0.58	0.02	5945
	8.41	100 000.00	0.6	0.59	0.01	11 891
Enoxaparin (40 mg)	156.00	100	0.6	−155.40	156.00	1
	156.00	500	0.6	−30.60	31.20	3
	156.00	1000.00	0.6	−15.00	15.60	6
	156.00	20 461.32	0.6	−0.16	0.76	131
	156.00	50 000.00	0.6	0.29	0.31	321
	156.00	100 000.00	0.6	0.44	0.16	641
Rivaroxaban (20 mg)	526.22	100	0.6	−525.62	526.22	0
	526.22	500	0.6	−104.64	105.24	1
	526.22	1000.00	0.6	−52.02	52.62	2
	526.22	20 461.32	0.6	−1.97	2.57	39
	526.22	50 000.00	0.6	−0.45	1.05	95
	526.22	100 000.00	0.6	0.07	0.53	190

Abbreviations: ARR, absolute risk reduction; INR, international normalized ratio; NNT, number needed to treat; VTE, venous thromboembolism.

**Table 4.** Sensitivity Analysis for Venous Thromboembolism Rate.

Drug	Cost of Drug, $	Cost of Treating VTE, $	Initial VTE Rate, %	Final VTE Rate, %	ARR, %	NNT
Aspirin (81 mg)	1.94	20 461.32	0.001	−0.01	0.01	10 547
	1.94	20 461.32	0.1	0.09	0.01	10 547
	1.94	20 461.32	0.6	0.59	0.01	10 547
	1.94	20 461.32	1	0.99	0.01	10 547
	1.94	20 461.32	3	2.99	0.01	10 547
	1.94	20 461.32	5	4.99	0.01	10 547
Aspirin (325 mg)	2.00	20 461.32	0.001	−0.01	0.01	10 231
	2.00	20 461.32	0.1	0.09	0.01	10 231
	2.00	20 461.32	0.6	0.59	0.01	10 231
	2.00	20 461.32	1	0.99	0.01	10 231
	2.00	20 461.32	3	2.99	0.01	10 231
	2.00	20 461.32	5	4.99	0.01	10 231
Warfarin (5 mg)	2.22	20 461.32	0.001	−0.01	0.01	9217
	2.22	20 461.32	0.1	0.09	0.01	9217
	2.22	20 461.32	0.6	0.59	0.01	9217
	2.22	20 461.32	1	0.99	0.01	9217
	2.22	20 461.32	3	2.99	0.01	9217
	2.22	20 461.32	5	4.99	0.01	9217
Warfarin (5 mg) + INR	8.41	20 461.32	0.001	−0.04	0.04	2433
	8.41	20 461.32	0.1	0.06	0.04	2433
	8.41	20 461.32	0.6	0.56	0.04	2433
	8.41	20 461.32	1	0.96	0.04	2433
	8.41	20 461.32	3	2.96	0.04	2433
	8.41	20 461.32	5	4.96	0.04	2433
Enoxaparin (40 mg)	156.00	20 461.32	0.001	−0.76	0.76	131
	156.00	20 461.32	0.1	−0.66	0.76	131
	156.00	20 461.32	0.6	−0.16	0.76	131
	156.00	20 461.32	1	0.24	0.76	131
	156.00	20 461.32	3	2.24	0.76	131
	156.00	20 461.32	5	4.24	0.76	131
Rivaroxaban (20 mg)	526.22	20 461.32	0.001	−2.57	2.57	39
	526.22	20 461.32	0.1	−2.47	2.57	39
	526.22	20 461.32	0.6	−1.97	2.57	39
	526.22	20 461.32	1	−1.57	2.57	39
	526.22	20 461.32	3	0.43	2.57	39
	526.22	20 461.32	5	2.43	2.57	39

Abbreviations: ARR, absolute risk reduction; INR, international normalized ratio; NNT, number needed to treat; VTE, venous thromboembolism.

**Table 5.** Sensitivity Analysis for Drug Cost.

Drug	Cost of Drug, $	Cost of Treating VTE, $	Initial VTE Rate, %	Final VTE Rate, %	ARR, %	NNT
Any drug	0.5	20 461.32	0.6	0.60	0.00	40 923
	1	20 461.32	0.6	0.60	0.00	20 461
	5	20 461.32	0.6	0.58	0.02	4092
	10	20 461.32	0.6	0.55	0.05	2046
	25	20 461.32	0.6	0.48	0.12	818
	50.00	20 461.32	0.6	0.36	0.24	409
	75.00	20 461.32	0.6	0.23	0.37	273
	100.00	20 461.32	0.6	0.11	0.49	205
	250.00	20 461.32	0.6	−0.62	1.22	82
	500.00	20 461.32	0.6	−1.84	2.44	41
	1000.00	20 461.32	0.6	−4.29	4.89	20

Abbreviations: ARR, absolute risk reduction; NNT, number needed to treat; VTE, venous thromboembolism.

In matched cohort subanalysis evaluating rates of bleeding events and transfusions, we found that patients who received VTE chemoprophylaxis had higher rates of postoperative transfusion (0.82% vs 0.25%, *P* < .001) and bleeding events (0.56% vs 0.26%, *P* < .001).

## Discussion

In this study, we found that aspirin 81 mg and aspirin 325 mg daily are both cost-effective for VTE chemoprophylaxis in patients undergoing operative fixation of an ankle fracture at the low, TriNetX, and high rates of symptomatic VTE. Warfarin 5 mg daily is also cost-effective at all 3 rates of symptomatic VTE, even when accounting for the cost of INR testing. Enoxaparin 40 mg daily is only cost-effective at the high symptomatic VTE rate. Rivaroxaban 20 mg daily is not cost-effective at any rate. Enoxaparin and rivaroxaban only become cost-effective at much higher costs of symptomatic VTE treatment ($50 000 and $100 000, respectively).

### VTE in Foot and Ankle Surgery Patients

This study found a VTE rate of 0.6% for patients undergoing operative fixation for foot and ankle fractures who were not on postoperative VTE prophylaxis. However, VTE rates in the literature vary greatly. Some studies have demonstrated very low incidence of venous thromboembolic disease in patients following foot and ankle surgery. A retrospective study of 45 949 ankle fracture surgery patients from the National Health Service patients in the United Kingdom demonstrated very low rates of DVT (0.12%), PE (0.17%), and mortality (0.37%) within 90 days of surgery.^11^ Additionally, a study by Marder et al^14^ found a VTE rate of 0.33% among patients following foot and ankle fracture surgery.

Conversely, other studies report VTE rates higher than the rate identified in our study. A recent prospective study using a UK national assessment tool demonstrated that the incidence of VTE events following ankle fracture fixation is 3.0% at a mean of 37.2 ± 14.2 days.^1^ Brennan et al^2^ identified a 1.2% VTE rate among 15 342 patients who underwent foot and ankle fracture surgery. Another prospective study of foot and ankle surgery patients demonstrated a 5.09% incidence of VTE postoperatively in patients who were placed in a cast postoperatively and instructed to maintain nonweightbearing to the operative extremity.^15^ As demonstrated in the literature, the reported VTE rate after foot and ankle fracture surgery varies because of the heterogeneity of procedures, differences in postoperative immobilization protocols, and variability in diagnostic methods for VTE detection. In the sensitivity analysis, we found that as the initial VTE rate increased, the higher-cost agents enoxaparin and rivaroxaban became cost-effective and had lower NNTs compared with the less expensive medications aspirin and warfarin. These findings indicate that in patients with elevated VTE risk, the use of more costly agents may be both economically justified and clinically appropriate, but must be weighed against bleeding risk, patient-specific contraindications, and limited data on clinical benefit.

### Risks of VTE Chemoprophylaxis

In a matched control subanalysis, we found that patients receiving enoxaparin demonstrated slightly higher rates of bleeding events and transfusions (<1%) compared with matched controls at 30 days postoperatively, whereas those receiving aspirin, warfarin, and rivaroxaban had comparable rates to matched controls. Previous studies have also demonstrated low incidence of harm from prophylactic dose anticoagulation. A systematic review by Horner et al^9^ of 7000 patients treated with Lovenox for VTE chemoprophylaxis found only 4 major bleeding events in total. Additionally, a meta-analysis of randomized controlled trials studying the efficacy of VTE chemoprophylaxis in patients following foot and ankle surgery found an overall incidence of major bleeding in this patient population of 1 in 886 (0.11%).

Conversely, some studies have shown that VTE chemoprophylaxis does not increase rate of bleeding complications. A case-control study of outpatients treated with plaster cast immobilization of the leg by Kock et al^12^ demonstrated no severe side effects of LMWH. A prospective, double-blind, placebo-controlled trial of 440 foot and ankle surgery patients by Lassen et al^13^ demonstrated no difference in bleeding events between patients receiving Lovenox for VTE chemoprophylaxis and the control group. A randomized, double-blind, placebo-controlled study by Lapidus et al^10^ demonstrated no significant difference in major bleeding events between patients receiving dalteparin for VTE chemoprophylaxis and the control group. Given the overall low rates of bleeding and transfusions in our study, along with mixed results in the literature, the clinical significance of these findings remains uncertain.

### Patient-Specific Approach

Despite meeting cost-effectiveness thresholds, aspirin and warfarin demonstrated extremely high NNTs, each exceeding 9000, which implies that a large number of patients must receive treatment to prevent a single adverse event, thereby diminishing the practical impact of the intervention. These findings emphasize that cost-effectiveness alone should not be interpreted as justification for routine prophylaxis. Instead, decisions regarding VTE chemoprophylaxis should consider individual patient risk factors, which is not accounted for by the cost-effectiveness model.

Surgeons may also need to consider differences among available VTE prophylactic agents. Aspirin, unlike other VTE prophylactic agents, is available over the counter, which may improve accessibility and reduce upfront costs for patients. However, its over-the-counter status may also introduce variability in adherence and dosing, as patients may not receive the same level of monitoring or guidance compared with prescription-based anticoagulants. These real-world implementation factors, such as patient compliance and appropriate use, are important considerations when determining the most suitable approach to VTE prophylaxis.

In determining whether to prescribe VTE prophylaxis, surgeons must weigh multiple patient-specific considerations. A recent study by Brennan et al^2^ demonstrated that risk factors for VTE in foot and ankle surgery patients were age ≥65 years, diabetes, dyspnea, congestive heart failure, dialysis, wound infection, and bleeding disorders. A patient’s individual risk profile can influence their likelihood of developing a VTE and may guide the decision to initiate chemoprophylaxis.

### Limitations and Future Research

This study has several limitations. This is a retrospective database study, so there exists some heterogeneity in the data collection methodology over time and throughout the various sites of the study. Additionally, there are many different types of ankle fractures with surgeon-specific treatment algorithms and unique postoperative rehabilitation protocol. This study did not include information on key factors such as pre- and postoperative radiographic findings, surgical techniques, injury mechanism, among others.

Additionally, the VTE incidence rates used in this analysis were derived from previously published database studies, which are subject to inherent limitations. Such studies may underestimate true VTE incidence because of reliance on diagnostic coding, lack of clinical adjudication, and underreporting of asymptomatic or delayed events. Additionally, database-derived rates may lack generalizability to specific patient populations or surgical settings, which introduces potential bias into the model assumptions. These sources are heterogeneous in design and may be subject to unmeasured bias, such as differences in coding practices or patient selection. No formal appraisal of study quality or coding validity was performed, which may affect the accuracy of incidence estimates and model assumptions.

Future studies should be done evaluating the cost-effectiveness of VTE chemoprophylaxis in other foot and ankle surgeries and the prescribing patterns of surgeons.

## Conclusion

In summary, this study found that aspirin 81 mg, aspirin 325 mg, and warfarin are cost-effective for VTE chemoprophylaxis following ankle fracture fixation. Enoxaparin and rivaroxaban are generally not cost-effective. Given the heterogeneity of ankle fractures with regard to the injury, surgery, and patient-specific risk factors for venous thromboembolic disease, no broad protocol exists that can be applied to all patients. These findings can inform decision-making but should not substitute for individualized clinical judgment. Cost-effectiveness alone does not justify routine use of prophylaxis in this population.

## Supplemental Material

sj-pdf-1-fao-10.1177_24730114251363497 – Supplemental material for Cost-Effectiveness Thresholds for Venous Thromboembolism Prophylaxis in Ankle Fracture Surgery: A Break-Even Analysis Supplemental material, sj-pdf-1-fao-10.1177_24730114251363497 for Cost-Effectiveness Thresholds for Venous Thromboembolism Prophylaxis in Ankle Fracture Surgery: A Break-Even Analysis by Kush Mody, Avani A. Chopra, David Ahn, Michael Aynardi and Sheldon Lin in Foot & Ankle Orthopaedics

## Appendix

**Supplemental Table 1.** *ICD-10* Coding for Comorbidities.

Condition	*ICD-10* Code
Acute myocardial infarction	I21
Congestive heart failure	I50
Peripheral vascular disease	I73
Cerebral vascular accident	I63
Dementia	F03
Pulmonary disease	J40-J4A
Connective tissue disorder	M30-M36
Peptic ulcer	K25
Liver disease	K70-K77
Diabetes mellitus	E08-E13
Hemiplegia	G81.9
Renal disease	N18
Cancer	C00-D49
HIV	B20
History of VTE	I26, I82.4
Antiphospholipid syndrome	D68.61
Hereditary factor VIII deficiency	D66

Abbreviations: HIV, Human Immunodeficiency Virus, *ICD-10*, *International Classification of Diseases, Tenth Revision*; VTE, venous thromboembolism.

## References

[1] Blanco JA Slater G Mangwani J. A prospective cohort study of symptomatic venous thromboembolic events in foot and ankle trauma: the need for stratification in thromboprophylaxis? J Foot Ankle Surg. 2018;57(3):484-488. doi:10.1053/j.jfas.2017.10.036 29503135

[2] Brennan J Keblish D Friedmann E Spirt A Holt E Turcotte J. Postoperative venous thromboembolism risk-prediction in foot and ankle fracture surgery. Foot (Edinb). 2023;56:102017. doi:10.1016/j.foot.2023.102017 36966559

[3] Chambers S Chadda S Plumb JM. How much does international normalized ratio monitoring cost during oral anticoagulation with a vitamin K antagonist? A systematic review. Int J Lab Hematol. 2010;32(4):427-442. doi:10.1111/j.1751-553X.2009.01205.x 19930411

[4] CPI inflation calculator. U.S. Bureau of Labor Statistics. Accessed April 16, 2025. https://www.bls.gov/data/inflation_calculator.htm

[5] Falck-Ytter Y Francis CW Johanson NA , et al. Prevention of VTE in orthopedic surgery patients. Chest. 2012;141(2 suppl):e278S-e325S. doi:10.1378/chest.11-2404 PMC3278063 22315265

[6] Griffiths JT Matthews L Pearce CJ Calder JDF . Incidence of venous thromboembolism in elective foot and ankle surgery with and without aspirin prophylaxis. J Bone Joint Surg Br. 2012;94(2):210-214. doi:10.1302/0301-620X.94B2.27579 22323688

[7] Grosse SD Nelson RE Nyarko KA Richardson LC Raskob GE. The economic burden of incident venous thromboembolism in the United States: a review of estimated attributable healthcare costs. Thromb Res. 2016;137:3-10. doi:10.1016/j.thromres.2015.11.033 26654719 PMC4706477

[8] Hatch MD Daniels SD Glerum KM Higgins LD. The cost effectiveness of vancomycin for preventing infections after shoulder arthroplasty: a break-even analysis. J Shoulder Elbow Surg. 2017;26(3):472-477. doi:10.1016/j.jse.2016.07.071 27727049

[9] Horner D Goodacre S Pandor A , et al. Thromboprophylaxis in lower limb immobilisation after injury (TiLLI). Emerg Med J. 2020;37(1):36-41. doi:10.1136/emermed-2019-208944 31694857 PMC6951266

[10] Lapidus LJ Ponzer S Elvin A , et al. Prolonged thromboprophylaxis with Dalteparin during immobilization after ankle fracture surgery: a randomized placebo-controlled, double-blind study. Acta Orthop. 2007;78(4):528-535. doi:10.1080/17453670710014185 17966008

[11] Jameson SS Augustine A James P , et al. Venous thromboembolic events following foot and ankle surgery in the English National Health Service. J Bone Joint Surg Br. 2011;93-B(4):490-497. doi:10.1302/0301-620X.93B4.25731 21464488

[12] Kock HJ Schmit-Neuerburg KP Hanke J Rudofsky G Hirche H. Thromboprophylaxis with low-molecular-weight heparin in outpatients with plaster-cast immobilisation of the leg. Lancet. 1995;346(8973):459-461. doi:10.1016/S0140-6736(95)91320-3 7637478

[13] Lassen MR Borris LC Nakov RL. Use of the low-molecular-weight heparin reviparin to prevent deep-vein thrombosis after leg injury requiring immobilization. N Engl J Med. 2002;347(10):726-730. doi:10.1056/NEJMoa011327 12213943

[14] Marder RA Danielsen B White RH Meehan JP. Incidence and time course of symptomatic thromboembolic outcomes after lower extremity arthroscopic surgery, ankle fracture surgery, and Achilles tendon repair. J Am Acad Orthop Surg. 2024;32(13):597-603. doi:10.5435/JAAOS-D-23-00495 38236919

[15] Saragas NP Ferrao PNF Saragas E Jacobson BF. The impact of risk assessment on the implementation of venous thromboembolism prophylaxis in foot and ankle surgery. Foot Ankle Surg. 2014;20(2):85-89. doi:10.1016/j.fas.2013.11.002 24796824

[16] Weisman MHS Holmes JR Irwin TA Talusan PG . Venous thromboembolic prophylaxis in foot and ankle surgery: a review of current literature and practice. Foot Ankle Spec. 2017;10(4):343-351. doi:10.1177/1938640017692417 28719780

